# Chicago Dental Society

**Published:** 1892-11

**Authors:** 


AMERICAN JOURNAL

—OF—

DENTAL SCIENCE.

VOL. XXVI. BALTIMORE, NOVEMBER, 1892. NO. 7.

ARTICLE I.

### CHICAGO DENTAL SOCIETY.

Regular meeting July 12, 1892, Dr. J. W. Wassall, president, in the chair.

Dr. E. A Royce read a paper entitled "Filling with Crystal Gold on the Surface of Amalgam."

The president called upon Dr. A. W. Freeman to open the discussion. Dr. Freeman said: I might say for once in this society you have taken me by surprise. I had no intimation that I should be called upon to open the discussion on this subject, as I have had very little experience with this method of making fillings. I therefore hardly know what to say. I feel that I cannot advise you as a good adviser. I should first like to see the specimens and then judge in regard to the work. I would say that I am always willing to try anything and everything that I think will save a tooth. I have always made it a practice of buying things at considerable expense oftentimes, and sometimes have found them of very little good. If we can judge a thing by its looks, I should think the specimen we have here might be very good. I have put in perhaps ten or twenty of these fillings, and they have extended over a period of four or five years. I have never used all crystal gold. I have used Steurer's gold, but did not have first rate success with it, and I have not had perfect success with crystal gold, as I should judge our friend has by his specimens. I have taken cases where I could hardly make a gold filling and have veneered the surfaces of them, and they remained quite well. I have oftentimes mingled with that a little phosphate, and I am looking for results from those fillings. I cannot say that I have had enough experience to recommend the use of phosphate and amalgam highly; but I have had enough experience to recommend it as a fairly good thing. I believe that it is better than all phosphates from my limited experience with it. I am here to gain knowledge, and not to impart much on this subject.

Dr. J. G. Reid.—Mr. President, I do not think I have very much to say on this subject. I have patched a few gold fillings with amalgam, or at least tried to, but have never had very much success with them. If I had taken the fillings out and filled the cavities anew, I perhaps would have been better off.

I attempted once to put in a gold and amalgam filling, and I got it about half-way in, then it all squeezed out and I quit. I went back to gold and tin, and finally filled it satisfactorily. I do not see that there is any particular advantage gained by using an amalgam filling over tin and gold. I believe a tin and gold filling combined will produce equally as good a result. I am not saying this from experience, because I am not experienced in the use of amalgam as Dr. Royce uses it. The amalgam filling I attempted to cover with foil I expect was a miserable failure, but it would not have been a success anyway. It looks well. I think the crystal gold combined with amalgam will look better than the foil, and I use crystalloid gold entirely, and have for years, and have not used anything else. I do not know whether crystalloid gold works with it nicely or not. It seems to me crystalloid gold would not work as well as Watt's crystal gold.

The specimen that has been passed around is very good indeed. All fillings look well out of the mouth. The only way to see them is in the mouth after they have been in two or three years. I should say it will expedite some fillings very materially, but not any more so than with tin and gold in combination. I have great confidence this combination and always have had. They fail sometimes just as any other filling, but that is not the question before the society. It is the question of amalgam and gold. I cannot speak on this subject from a practical standpoint. I have never put in one of these fillings in my life, and have never seen one put in as recommended by Dr. Royce—that is, the filling being started on soft amalgam.

Dr. C. P. Pruyn.—Unfortunately, I did not hear all the paper. I think I heard enough of it to get an idea of what the essayist intended to say. I have heard him talk on a previous occasion on this subject. I have tried in one or two instances to carry out the ideas that he has advanced, but I have not succeeded for some reason. I do not know just why I have been unsuccessful, but I have failed to make the gold work. I have failed to get the gold to thoroughly unite with the amalgam, and it became a mixed-up mass and did not work well in my inexperienced hands. The other method, the mechanical union of gold and amalgam, is one that has interested me considerably, but I think perhaps that is foreign to the subject of the paper. If it is not, I might talk a little on it; I am a thorough believer in gold and amalgam. I have practiced this method in many cases ever since I commenced to practice, putting amalgam in at one sitting, and at a subsequent occasion putting on gold. Where this is done there is a rapid oxidation of the amalgam, so that it becomes black like copper amalgam, and the gold remains bright and clear, and if proper pains are taken the amalgam filling is quite as good as with gold. In the worst cases, where you use such a combination, you preserve the teeth better than with either one of the metals alone. This is my belief, founded on fifteen or eighteen years' experience. Doubtless many of you have seen cases that have been in my hands in former years; they have drifted into your hands. You may have seen these operations. If you have not, I would like to show them to you. Take the class of teeth spoken about so persistently a few years since by Drs. Chase and Palmer, below the average in quality, they could not stand well with metal alone. If you take this combination, you will have better results with it, I think, than with any other one method that can be used. The curse that was heaped upon amalgam ought not to have been upon amalgam *per se*, but upon the slovenly way in which it has been used. It is a good filling material for saving teeth. There is none better if it is used as it should be. If any of you have never filled a glass tube with amalgam, do it, with paper wrapped around it; then take the paper off and see the imperfections in the filling, unless more than ordinary care has been used. The practitioner will see air spaces where he thought the amalgam was in complete opposition. It takes no longer to fill a tooth with amalgam now than it did fifteen years ago. I have found that it needs time, it cannot be done as we were instructed to do it years ago. It cannot be done slovenly; we must use just as much care and thoroughness as with any other material, and I don't know but more. Then, we have with it a peculiar electricial condition of things that will destroy the microbes that produce decay. Amalgam does its best work at the cervical margin; gold does its poorest work at the cervical margin. Amalgam does its poorest work upon crown surfaces or angles; gold does its best work there. Take a large cavity in the distal surface of a second molar, fill it with amalgam, but with no attenuated edges. The cavity must be prepared differently for gold. We must have good, sharp, square-cut walls; otherwise the attenuated walls would be clipped down and break off. We must have a beveled surface; there you use gold to advantage, using these two metals only. Where you use one at its strongest point and where it does its best work, you do your patient better service than if you attempt to use gold altogether. When I do work of this kind, I do not propose to put in such fillings for $1.50 or $3.00. If necessary, I charge $10.00; I charge the patient for time. If you are doing your patients a service with amalgam that you could not do for them with gold, charge them for it just as though you were using gold.

Dr. J. H. Woolley.—In using a combination of amalgam and gold, do you fill with gold before the amalgam sets?

Dr. Pruyn.—I do not. I have never advocated that system. It does not seem to me that it is as practical, when done on mechanical and philosophical principles, as the other; still the essayist has shown from his experience that it is satisfactory in his hands.

Dr. A. E. Matteson.—I cannot say that I have had a great deal of experience with amalgam and gold as a filling material. I am firmly convinced that amalgam is a good thing, and that gold is a good thing. I have seen some very poor results from the use of amalgam and gold in combination from the best operators in the country. I have used amalgam and gold in combination frequently in repairing cervical margins, and I have had very good results following the use of them in that way. The great trouble with the use of amalgam is, in my experience, due to a lack of proper manipulation. I doubt if twenty-five per cent. of practitioners who are using amalgam use it as it ought to be used. They do not follow the directions. They have not the combination that was originally made, because they make amalgam and press out parts of the alloy with the mercury in excess. It is all wrong. If the combination is correct it should be there, and the mercury should be in proportion, as it forms the mass. If there is too much mercury, add more alloy, and *vice versa.* I believe that a great majority of the failures are from a lack of manipulation. I have occasionally filled cavities (proximal) with the first half of amalgam, and gold for the remainder, for a number of years, allowing the amalgam to remain two or three days, then polishing and finishing it, then building over it with gold. I believe that there is chemical union after it has been allowed to set.

Dr. A. W. Harlan.—I listened very attentively and carefully to Dr. Royce's paper, and his ideas on the subject are very explicit. He says in a certain class of cases he mixes the amalgam dry and uses a matrix, packs the amalgam between the cavity wall and matrix nearly up to the edge of it and adds crystal gold until there is perfect union, then he fills the tooth with gold. That is very plain, and I believe I will try it. I have filled a great many teeth by introducing amalgam first, then cutting out the next day or at some other period a sufficient quantity and welding on with gold. I think it is justifiable practice, but not always. I tell the patient what I do it for. I have had very good results from operations of this kind, but to say that it should become a general practice would be far from what I mean. It is adapted for a certain class of cases where it is impossible to make a good, firm joint between the gold and tooth structure on account of the difficulty of reaching it with gold, so that it can be thoroughly packed, and the class of teeth where there is still greater difficulty in making a perfect edge out of the quality of the material. This other method, it seems to me, is preferable to the one I have practiced. I shall try it. Anything that will help to save a tooth is worthy of trial, and this seems to offer a new line for experiment. You do not need to make many of these fillings before you find out what the result will be, and if it is satisfactory it will become an accepted mode of practice.

Dr. R. B Tuller.--My first experience in trying the method of filling outlined by Dr. Royce was a little peculiar. I heard him describe his method on a previous occasion, and the first opportunity I had I tried it. I put in my amalgam at the cervical margin, filled up about one-third, and commenced packing in Watt's crystal gold, and as I was packing that in I was knocking out the amalgam, until I finally found the cavity was filled entirely with gold. That experience taught me that the matrix was an essential feature, and since that I have tried it in several cases where it has given me a great deal of satisfaction; but whether it will stand the test of time we will have to wait and see. It has been so satisfactory to me in a certain class of cases, as described by Dr. Royce, that I shall try it, I think, whenever such cases present; but I am an advocate of gold wherever I can use it and make a filling that will do what we aim to do—preserve the teeth. There are some cervical borders where the best and most skillful gold-workers in the profession could not, in my estimation, make as good a gold filling as could be done with amalgam.

Dr. Geo. J. Dennis.—I have had no experience in this line, but I have had the opportunity of seeing several of Dr. Royce's fillings that have been in four or five years, and all of them seem to preserve the teeth, with the exception that the walls of the teeth were frail; they were split off, leaving the gold and amalgam exposed from the crowns of the teeth to the cervical border. When I looked at the tooth, it seemed to me the wall had broken down, or the tooth had split off because of the expansion of the metal. Whether that was true or not I cannot positively say. There appeared to be a chemical union between the two metals, the gold and amalgam. The amalgam was pitted on its surface. The proximal surface was also pitted to a certain extent on the surface next to the tooth wall. The only objection I have to the use of amalgam is, that there is an unequal degree of expansion or shrinkage in the case of the gold; that is, if very much mercury is used in the amalgam it takes up considerable gold. In the case in which the wall was split off the cervical border was perfectly preserved.

Dr. J. N. Crouse.—I have not had much experience with a combination of amalgam and gold, except as I have observed them in the mouths of patients, or where a gold filling had been put in and somebody put amalgam in and patched it up. I had a mouth of that kind to-day with eight or ten amalgam fillings combined, in different teeth. From the hands the cases were in, I know why amalgam was in the teeth. In one or two instances I have gouged out some portions of amalgam fillings and added gold rather than take out all the amalgam. It is a kind of operation that I should regret to see highly recommended to beginners or practitioners in middle life or old age. It tends, I think, to slovenliness. You can put an amalgam filling in a tooth where it is not half prepared and have it look well, and the chances are that if you operate with amalgam you will not properly prepare the cavity, whereas you would if you used gold. It has a tendency also, in my opinion, to take away the enthusiasm of the operator, that influence that every good operator must have and that fills him with encouragement, when he has done a good piece of work. When a dentist has performed a good operation he looks upon it with pleasure. One of the fascinations of operative dentistry is the beauty and perfection the operator sees after he has made a great effort. It is not only true of dentistry, but it is also true of everything in which a man succeeds.

What can be the advantages of amalgam and gold combined? It has been said that it has an additional influence in that it destroys microbes That is a very indefinite proposition. If there is anything in a combination of mercury and tin, platinum and copper fillings, of amalgam and gold, I should think it would be used exclusively for the destruction of microbes. Personally I have very little faith in such a theory or that kind of recommendation. The only good ground for the use of this combination is where the cavity passes so high up that it is impossible to get the dam above the cervical margin without inflicting a great amount ef torture to the patient. You can pack amalgam quicker, and the very small amount of moisture that comes in contact with it and the cervical margin will not interfere with the operation. I can see how that can be done with credit and success. Generally speaking, if the practitioners of dentistry would practice the old and first method that had merit in it, which is packing soft cylinders of non cohesive gold, they would have an operation that is more perfect as to the safety of a tooth, consume very little more time, and would have that influence that shows when you are through that you have accomplished the object that you started out to accomplish. Show me any college that teaches its pupils how to pack soft cylinders of non-cohesive gold, and I would like to have a photograph of it. If there is one in this country that does it, I do not know where it is. It requires good judgment and self-collection to do it. You cannot pack non-cohesive cylinders and talk; you have got to have your mind on the work. If there is any better way of filling teeth than that, I want to see it.

If I understood the essayist correctly, one of his reasons why amalgam is better was that the packing of gold took away the excess of mercury, etc. I want to say that teeth can be saved in the way described by the author of the paper, but where are the advantages? Is it better than gold? I challenge that proposition to any kind of test that may be brought out. If you are going to use amalgam in connection with gold, I would recommend its use in large cervical cavities where decay has taken place beyond, and in order to fill such teeth with gold properly you have got a good deal to do. It would be justifiable to pack gold with amalgam in such places. I have performed this operation, I have gouged out gold away up under the margin of the gum, and have patched it with amalgam or gutta-percha. I do not know which is the better of the two, but I should say gutta-percha.

Dr. C. F. Hartt.—I believe most of the gentlemen who have spoken to-night are away off. I have given up entirely the filling of large cavities with metal of any kind. A tooth of good structure should be filled with gold, and teeth below the average should be filled with cement. If we want something to hold the outside walls of the cavity together, fill the teeth with cement, then cut out say the sixteenth or eighteenth of an inch all around up to the enamel margin, and have a smooth, clean cut-margin wherever the enamel and cement come together. Fill that full with gold. You cannot get up there with cylinders, a spongy mass like that. You make a little retaining point or groove, you can fill it with cement, and you will be astonished how little gold is necessary to spread over the surface to protect the cement. It is a mistake to fill teeth with metal of any kind. Cement is the thing: then pack over it anything you want to that is durable.

Dr. E. A. Royce.—One of the first objections that I expected to hear has not been spoken of; that is the liability to mistake the color given by the amalgam at the gum margin for decay. This may be obviated by allowing the amalgam to extend farther down upon the lingual aspect of the tooth so it is plainly visible.

In regard to non-cohesive gold, I think that since I have been in Chicago, I have given more clinics to demonstrate its use than any other man in the city. I have at the present time under observation a number of cases where the compound proximate cavities of molars and bicuspids are built up entirely of non cohesive. gold; the contour is such as to give fairly good points of contact, and the fillings are doing good service. I use the combination of tin and gold for a large class of cases, and am perfectly satisfied with it in its place.

Gold and amalgam should not take the place of gold. The best operators in the country are losing gold fillings every day; not entirely because of faulty manipulation, but in a great degree because of galvanic action. In the combination the amalgam will oxidize rapidly, stop the current, stop any minute openings, and in that way assist in saving the tooth. I do not attempt to save time by its use, but save the tooth that is below grade.

Crystal gold acts so nicely with mercury because it is manufactured by making a gold amalgam, and the mercury is then removed by acids, leaving the gold to be prepared for market.—*Dental Review.*

---

ARTICLE II.

